# TracMouse: A computer aided movement analysis script for the mouse inverted horizontal grid test

**DOI:** 10.1038/srep39331

**Published:** 2016-12-16

**Authors:** W. Niewiadomski, E. Palasz, M. Skupinska, M. Zylinski, M. Steczkowska, A. Gasiorowska, G. Niewiadomska, G. Riedel

**Affiliations:** 1 Mossakowski Medical Research Centre Polish Academy of Sciences, Warsaw, Poland; 2 Warsaw Medical University, Warsaw, Poland; 3 Nencki Institute, Warsaw, Poland; 4 Warsaw University of Technology, Warsaw, Poland; 5 Institute of Medical Sciences, University of Aberdeen, UK

## Abstract

In rodents, detection and quantification of motor impairments is difficult. The traction test (inverted grid with mice clinging to the underside) currently has no objective rating system. We here developed and validated the semi-automatic MATLAB script *TracMouse* for unbiased detection of video-recorded movement patterns. High precision videos were analyzed by: (i) principal identification of anatomical paw details frame-by-frame by an experimentally blinded rater; (ii) automatic retrieval of proxies by *TracMouse* for individual paws. The basic states of Hold and Step were discriminated as duration and frequency, and these principle parameters were converted into static and dynamic endpoints and their discriminating power assessed in a dopaminergic lesion model. Relative to hind paws, forepaws performed ~4 times more steps, they were ~20% longer, and Hold duration was ~5 times shorter in normal C57Bl/6 mice. Thus, forepaw steps were classified as exploratory, hind paw movement as locomotive. Multiple novel features pertaining to paw sequence, step lengths and exploratory touches were accessible through *TracMouse* and revealed subtle Parkinsonian phenotypes. Novel proxies using *TracMouse* revealed previously unidentified features of movement and may aid the understanding of (i) brain circuits related to motor planning and execution, and (ii) phenotype detection in experimental models of movement disorders.

The detection and experimental quantification of mouse motor impairments that truthfully mimic the anomalies of Parkinsonian patients has proved difficult. One of the most reliable means for the induction of extensive loss of dopaminergic neurons in the substantia nigra pars compacta is the systemic administration of 1-methyl-4-phenyl-1,2,3,6-tetrahydropyridine (MPTP) in both rodents and primates alike^1^,^2^. In contrast to robust pathological phenotypes following MPTP exposure, deficits in movement and motoric actions are hardly discernable in mice engaged in simple motor tasks, even though frank cell loss in the substantia nigra may level at >80%^3^,^4^,^5^. While a comprehensive analysis concerning the underlying reasons remains elusive, there are clear differences between central control of locomotion in quadrupedal vs. bipedal animals. In rodents (incl. mice), the basal ganglia exert a strong control over different aspects of forepaw movement and do much less so for hind limb activity^6^. As a corollary, the selection of motor tasks critically depending on forepaw manipulation should readily reveal and correlate with dysfunctional striatal dopamine.

Grip coordination tasks are amongst the most suitable tests to distinguish front and hind paw ataxias and include the ‘traction test’^7^, which consists of a horizontal wire grid to which mice are hung by their forepaws. Alternative methods utilized a vertical rod called the ‘string test’^8^, or a vertical grid ‘grip test’^9^. Typically, the time which mice cling to the wire and the quality of the grip are rated; however, even bradykinetic mice show strong clinging and a wide behavioral repertoire. Therefore, some standardization is needed^10^ and Tillerson and co-workers developed a new classification scheme for the traction test, also termed ‘inverted grid test’ (for detailed methodical description, see ref. 11). A mouse is placed on to a rectangular wire mesh grid with a wire distance of 0.5 cm and the grid is turned upside down, so the mouse is hanging down clinging on to the wire. Tillerson and colleagues video-taped the movement and visually extracted 3 proxies: Average Forepaw Step Distance, Percent Wall Time and Percent Forepaw Faults. In applying a two stage classification procedure, movement was first categorized as locomotive (active propulsion of paws to move forward is termed ‘step’) and non-locomotive (shuffling of paws across the grid, without real initiation of movement). Steps were further denominated as successful or unsuccessful such that movement of a paw to another area and placement with fingers around the grid constituted the former, while slipping or a failure to place the paw at a new grid location constituted the latter. From these proxies, the Percent Forepaw Faults was calculated as a ratio of unsuccessful and total number of attempted forepaw steps. The Average Forepaw Step Distance was derived from successful steps only. Unrelated to these measures was the Percent Wall Time when either head or trunk of the body made physical contact with the surrounding walls.

According to Tillerson *et al*.^12^ the three measures of inverted grid performance were superior in their sensitivity to reveal the effects of MPTP on forepaw movement in retired breeders of C57BL/6 mice; deficits were more robust and correlational compared to activity monitoring, Rotarod, and forepaw stride length during walking. Amongst the correlations, Tillerson and Miller^11^ detected sustained behavioral deficits up to 28 days post-injection in Average Step Distance, Percent Forepaw Faults and Percent Wall Time, the latter being positively correlated to doses of 7.5 or 15 mg/kg MPTP. Some recovery of the Average Step Distance and Percent Wall Time in these animals was revealed following L-DOPA administration or exercise^11^,^13^. The somewhat better performance in these treatment groups over MPTP alone significantly correlated with content of striatal dopamine (DA), dopamine transporter (DAT), vesicular monoamine transporter 2 (VMAT2) and tyrosine hydroxylase (TH) suggesting that recovery in these neurochemical markers is sufficient for treatment-related improvement in motor function. In reverse, Tillerson’s methodical 2-stage classification of movement may provide the sensitivity required to detect even subtle dopamine loss underpinning anomalies in motor function^11^,^12^,^13^.

A difficulty inherent to this approach is the subjectivity with which data were categorized. They required a “rater experienced in behavioral analysis of rodents”^11^, and especially the successful/unsuccessful classification is prone to failure. However, if this rating becomes ambiguous, not only does the parameter of Percent Forepaw Faults but also Average Forepaw Step Distance collapse and this would render the whole approach questionable. Thus, a more objective analysis tool with rater-independent settings is required. We here report such a method. In preparation of this work, we conducted a careful Web of Science analysis of all the work that applied the Tillerson and Miller^11^ analysis. From a total of 48 publications, 18 reported on the traction test (inverted grid), but the authors of 10 reports limited their analysis to hang time/cling time as an unambiguous simple index. Consequently, there is complete loss of information on the quality of the forepaw movements. As for the remaining 8 publications, there are gaps in the description of their methods, and some proxies are frequently omitted from the analysis without providing proper reasoning^14^,^15^,^16^,^17^,^18^,^19^,^20^,^21^.

Our approach did not aim at replicating Tillerson and Miller^11^ but was guided by their theoretical consideration of how movements on the inverted grid are executed. The core feature of our analysis became the ‘Hold’ state, in which there was no movement and animals maintained their grip statically in the same location. From high resolution video images, an experimenter marked the paw position during ‘Hold’ frame by frame on screen, extracted hold-related parameters and auto-analyzed any movement related shift of the paw using a MATLAB based script. This enabled unbiased detection of forepaw/hind paw activity. We also included a small cohort treated with MPTP to confirm phenotypes reported in the literature, and to reveal the sensitivity of the new analysis tool TracMouse qualitatively.

## Results

### Cohort selection and exclusions

Four control and six MPTP mice were exposed to the traction test at 10 and 20 days post treatment. In the first session all mice except two MPTP subjects were able to sustain 30 s on the grid. The two mice lasted no longer than 4.3 s and 10.5 s, respectively, and this were best results out of three consecutive trials. These same mice completed 30 s on the grid during the second session. This seems to suggest that they were under the early influence of MPTP at session 1 and recovered during the interim period before session 2. Nevertheless, their data from both sessions were omitted from the analysis, as too few samples of each proxy (see Tables 1 and 2 for detail) were available from the first session.

In the second session two other mice fell off the grid after 15.4 s (control mouse) and 16.2 s (MPTP mouse), despite a 30 s cling time in session 1 for both subjects. In this case, we saw no reason for exclusion based on treatment; moreover, we were able to retrieve enough samples for all proxies being calculated and data from these animals were included in the analysis after normalization. Consequently, data from 4 MPTP and 4 control mice were considered for the analysis.

### Cling Time

The values of Cling Time are detailed in Table 3. Since they did not match the cut-off of 30 s due to the physical process of terminating the trial our recordings exceeded this cut-off. The only value in which accurate frame-by-frame recordings were possible were trials in which animals pre-maturely fell off the grid. This global inaccuracy introduced a variable error <10% of the nominal 30 s. Furthermore, in cases when ‘Cling Time’ was substantially shorter, all proxies directly dependent on trial duration would be skewed. Consequently, a normalization procedure i.e. multiplication by Correction Factor was applied (see Table 3 for details).

**Figure 1:**
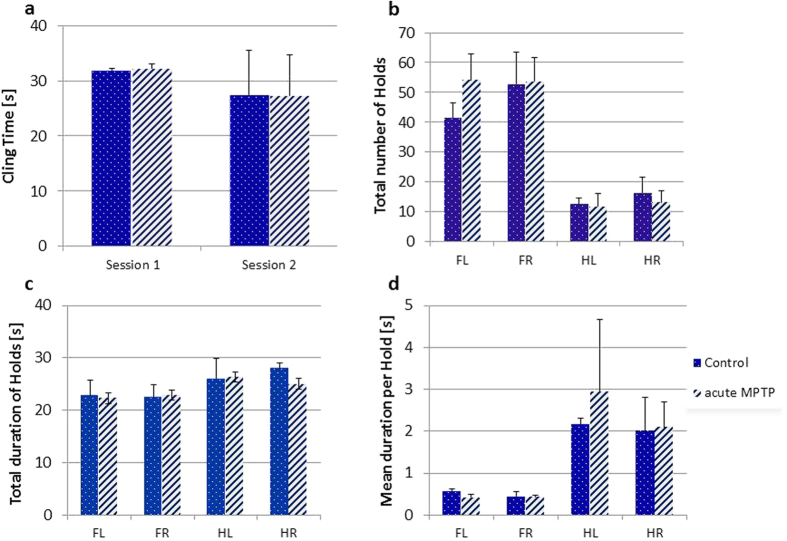
Mean values (±SD) of hold-related indices in C57BL/6 saline and MPTP treated mice. **(a)** ‘Cling Time’[s] was very similar for Session 1 and also for Session 2 and no statistically meaningful difference between vehicle and MPTP treated groups was detected. Note that for a comprehensive analysis of all movements of the traction test a total of >15 second cling time was required. Two subjects in the MPTP group did not fulfill this criterion and were excluded from the analysis. **(b)** ‘Total number of Holds’ was significantly greater for left (FL) and right forepaws (FR) relative to left (HL) and right hind paws (HR) in mice from both groups. MPTP mice performed significantly more holds with forepaws than the controls (see text for details). **(c)** ‘Holds duration’ [s] showing the cumulative time spent by a paw holding the grid was significantly shorter in forepaws than in hind paws both in saline and MPTP groups. **(d)** ‘Mean duration per Hold’ [s] was significantly shorter in forepaws than in hind paws in both groups.

As seen from the comparison of the data of 4 controls and 4 MPTP treated mice (Fig. 1a), means are very similar for Session 1 and also for Session 2. No statistically meaningful difference between means was detected, both within Session–Controls vs. MPTP mice as well as within Groups–Session 1 vs. Session 2. Consequently, all data presented here constitute averages between Session 1 and Session 2 rather than giving preference to one session over the other.

### Novel indexes of the traction test

#### Hold

The most important primary outputs from our novel analysis are ‘Total number of Holds’ (Fig. 1b) and ‘Hold duration’ (Fig. 1c). These are presented in a paw-specific manner, but we have not yet found evidence for any lateralization of movement. Therefore, it is suggested that data from front and hind paws may be merged. For the purpose of introducing these novel read-outs, however, we present them for each paw individually.

Both number and duration of Hold give precise information of the overall activity of an animal while clinging to the grid. This is different from the overall Cling Time as the latter also incorporates paw displacements as long as they do not lead to the animal falling down altogether. Thus, Hold provides a higher precision of the overall periods of inactivity. Total number of Holds (Fig. 1b) was clearly higher in front paws (significant main effect of front/hind paws (FH) factor, F(1,6) = 503.6, p < 0.00001). This already suggests that some exploratory movement is performed by the front paws while hind paws remain stationary for longer and display fewer displacements (Fig. 1b). Intriguingly, we found some evidence for an MPTP effect, since treatment and paw axis interacted (F(1,6) = 7.1, p < 0.05). Although not reliably different in simple t-test comparisons, this interaction appears due to a greater number of forepaw holds in the MPTP cohort (control 47.1 ± 7.1 vs. MPTP 53.8 ± 6.4) while their hind paws displayed fewer holds (control 14.4 ± 3.4 vs. MPTP 12.3 ± 3.9).

Although not striking, the cumulative duration in Hold was longer for hind paws in controls (fore: 22.6 ± 1.8 s; hind: 26.3 ± 2.3 s) and MPTP treatment groups alike (fore: 22.6 ± 1.3 s, hind: 25.7 ± 2.1 s). This explains the main effect of paw axis (F(1,6) = 51.4, p < 0.0005), and the lack of significance for the factor treatment.

A direct corollary of the above endpoints is that the average duration of a single Hold (‘Mean duration per Hold’) (Fig. 1d) was significantly shorter for forepaws than for hind paws (F(1,6) = 39.5, p < 0.001). Again, treatment had no effect on this proxy.

#### Steps

**Figure 2:**
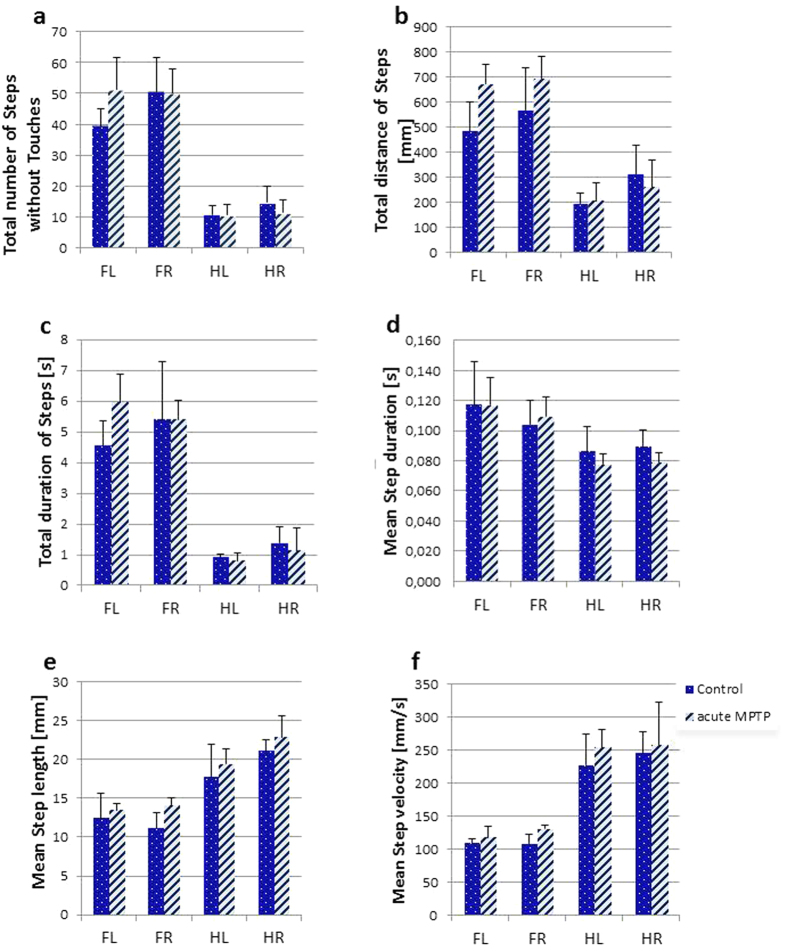
Mean values (±SD) of step-related indices in C57BL/6 saline (control) and MPTP treated mice. **(a)** ‘Total number of Steps without touches’ was significantly greater for forepaws (FL, FR) than for hind paws (HL, HR) and this was similarly observed in both test groups. **(b)** The total distance covered by a paw during the trial - ‘Total distance of Steps’ [mm] - was significantly higher for front paws and treatment interacted significantly with front/hind paws as factor. This was a result of greater distance covered by forepaws in MPTP mice. **(c)** ‘Total duration of Steps’ was significantly longer for forepaws compared with hind paws and this applied to both test cohorts. **(d)** ‘Mean duration of a Step’ [s] was significantly longer in forepaws than in hind paws. **(e)** ‘Mean length of each Step’ [mm] was significantly longer for hind than for forepaws as was the ‘Mean Step velocity’ [mm/s] **(f).**

From our algorithm, it is clear that the values for ‘Total number of Steps without Touches’ are, by definition, highly similar to the ‘Total number of Holds’ (Fig. 2a). Significantly more Steps were performed by forepaws than by hind paws (main effect paw axis F(1,6) = 334.6, p < 0.00001) and this was similarly observed in MPTP treated mice. Consequently, there was no significant difference for the factor treatment.

The total distance covered while moving on the underside of the grid - ‘Steps distance’ - was significantly higher for front paws (F(1,6) = 177.0, p < 0.00005) because of a higher number of exploratory short-distance movements (Fig. 2b). The factor treatment interacted significantly with paw axis (F(1,6) = 11.0, p < 0.05) possibly owing to the greater distance covered by forepaws of MPTP mice, which also differed between groups (p < 0.05) while the distance covered by hind paws was similar between groups. From these data, it is intuitively clear that while hanging from the grid, there is a great deal of front paw movements not combined with a displacement of the hind paws. Such movement is exploratory and it appears that MPTP mice show a greater propensity for exploration than the saline controls.

The cumulative time executing steps – ‘Steps duration’ - is shown in Fig. 2c and was significantly longer for forepaws compared with hind paws (F(1,6) = 284.0, p < 0.00001). This applied to both test cohorts (saline and MPTP) and there was no difference between these groups.

Consequently, we concentrated on a more in-depth definition of the characteristics of each step (Table 1).

The average duration of each step – ‘Mean Step duration’ – (Fig. 2d) was significantly longer in forepaws (F(1,6) = 42.7, p < 0.001). MPTP treatment had no significant effect. At the same time, the average length of each step – ‘Mean Step length’ – (Fig. 2e) was clearly longer for hind paws (F(1,6) = 128.6, p < 0.00005) and these were executed at higher velocity – ‘Mean Step velocity’ – (Fig. 2f) (F(1,6) = 118.7, p < 0.00005).

#### Touches and Wall Contacts

**Figure 3:**
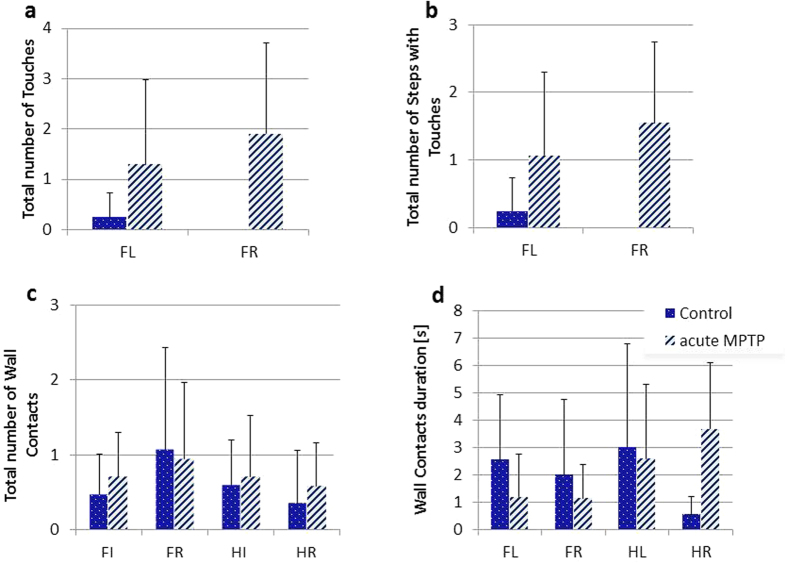
Mean values (±SD) in C57BL/6 saline and MPTP treated mice of: **(a)** ‘Total number of Touches’ performed during the trial by forepaws (FL, FR) was distinctly greater in MPTP treated mice. **(b)** ‘Total number of Steps with Touches’ in MPTP mice also exceeded the number observed in controls. **(c)** ‘Total number of Wall Contacts’ shows the number of contacts performed by fore and hind paws (HL, HR) during nominal trial. **(d)** ‘Wall Contacts duration’ shows the cumulative time spent in paw contact with wall.

During the movement on the underside of the grid, we observed a number of touches which did not lead to a hold but interrupted the steps and were followed by further movements. These touches were only conducted with forepaws. Our analysis retrieved two proxies for this behavior: ‘Total number of Touches’ (Fig. 3a) and ‘Total number of Steps with Touches’ (Fig. 3b). Both returned similar values as we found that in most cases only one touch interrupted a step. The overall incidence of touches occurring in control mice was very small; only 1 single mouse presented with 2 touches during the trial and we assume that normal mice do not usually execute touches while clinging and moving on the underside of a grid. In contrast, three MPTP treated mice repeatedly presented touches to the grid (total for both front paws in session 1 and 2: 27); the forth mouse showed no touches at all. Clearly, the lack of touches in controls precludes a statistically meaningful analysis and reveals that although some proxies are missing in normal animals, a disease-specific appearance may be registered. It therefore seems that disturbances in motor planning, which is typically observed in Parkinson patients^22^, can be determined based on the precise execution of movement at the underside of the grip. Such parameters are difficult to establish using conventional motor tasks.

On few occasions, we observed steps along the edges of the grid and animals executing wall contacts with their paws. These were found independent of paw axis and it appears that mice do not prefer execution of wall contacts with front over hind paws. The ‘Total Number of Wall Contacts’ per trial (Fig. 3c) is very low and was not different between treatment groups. In contrast to touches, which were very brief, the time spent by paws in contact with walls – ‘Wall Contact Duration’ – (Fig. 3d) constituted a significant amount of the total trial duration (up to 10%) and was exceeding the cumulative step time. It may thus be prudent to consider Wall Contacts as specific/anomalous Holds and not as part of the movement, i.e. Steps. The fact that treatment interacted with paw axis in the analysis of the ‘Wall Contacts Duration’ (F(1,6) = 7.74, p < 0.05) is probably due only to the short right hind leg duration in controls but our *Post hoc* analysis did confirm that this was the source of significance.

#### Regularity Index

**Figure 4:**
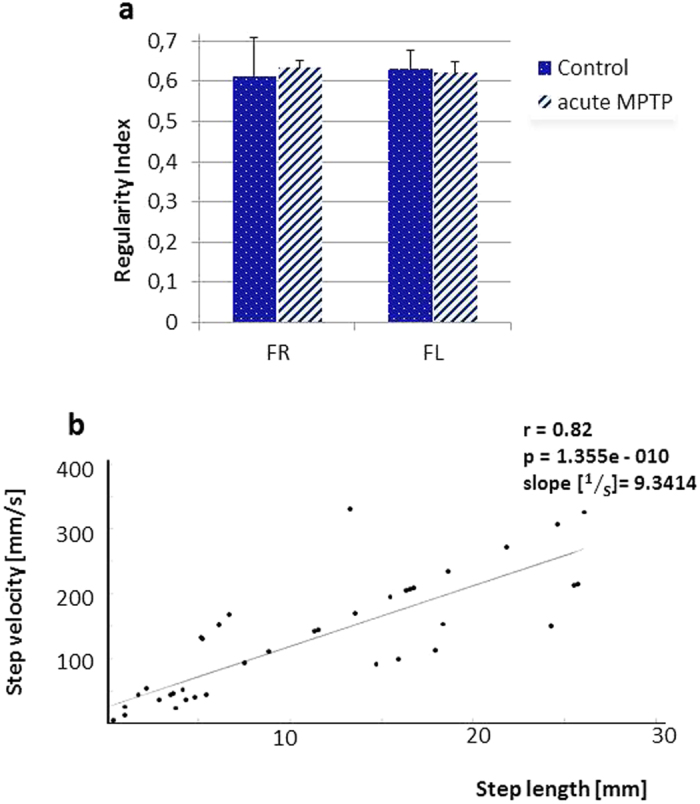
(**a**)‘Regularity Index’ calculated from long-short-long step sequences (compare Fig. 8). The overall value of the Regularity Index it unaffected by treatment and laterality (mean ± SD). **(b)** The correlation between step length and step velocity yielded highly significant correlations such that long steps are executed with higher velocity than shorter steps. The 9.34 1/s slope calculated from the example indicates that an increase of 10 mm step length would be matched by an increase in velocity of 93.4 mm/s.

If one considers all steps in a trial for each forepaw, it appears that these follow the pattern of longer-shorter-longer steps. Overall, the step length was at least twice as long in long steps relative to preceding short steps (data not shown). From this step pattern, we calculated the ‘Regularity Index’ (RI) as the amount of peaks in all steps of each session. Typically, 2–3 steps were performed with the right forepaw and then the subject switched sides and continued moving the left forepaw for several steps, before reverting to the right forepaw. A corollary of this motor program is that the use of one paw is discontinuous but the pattern of long-short steps remained intact despite these interruptions. Regularity Indexes for control and MPTP cohorts are displayed in Fig. 4a. Intriguingly, there was a similar level of alternation in all animals and this was not affected by treatment, as the mean values for left and right forepaws in controls and MPTP mice were virtually identical (all F’s < 1).

We also explored the relationship between velocity and step length and returned a significant positive correlation such that longer steps were typically undertaken at higher speed compared to shorter steps (Fig. 4b).

### Nigral DA cell loss following MPTP treatment

**Figure 5:**
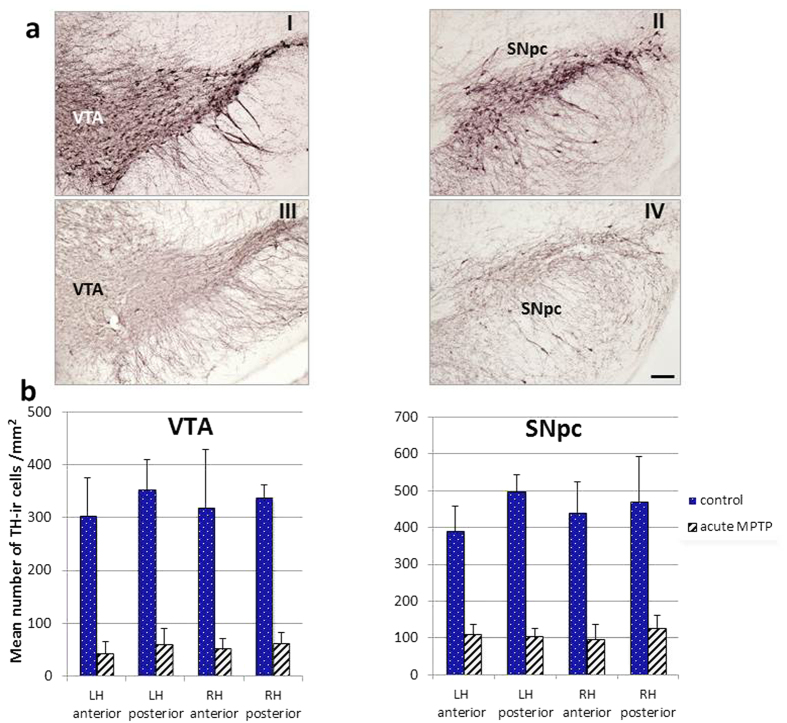
Histological and quantitative analysis of frank dopamine cell loss in MPTP mice (n = 6) compared to control (n = 4). **(a)** Microphotographs of the ventral tegmental area (VTA, I and III) and the substantia nigra pars compacta (SNpc, II and IV) in vehicle control (I and II) and MPTP treated mice (III and IV). **(b)** Mean packing density (as a number of cells/mm^2^) (±SD) of TH-immunoreactive neurons in the ventral tegmental area (VTA, left graph) and the substantia nigra pars compacta (SNpc, right graph) comparing vehicle controls and MPTP mice with acute treatment. Package density was calculated separately in both hemispheres as well as in the anterior and the posterior part of VTA and SNpc. Bar = 100 micrometers.

We distinguished between left and right hemisphere and the anterior and posterior portion of each region of interest. Immunhistochemistry (Fig. 5a) and cell counting (Fig. 5b) confirmed that the number of nigral dopaminergic neurons in the mice injected with MPTP was reduced relative with vehicle controls. This also lowered the mean packing density of TH-ir cells in the MPTP group. The loss of TH-ir neurons in MPTP mice was most pronounced in the VTA where an 84.0% reduction in immunolabeled cells was observed (Fig. 5 aI and aIII; Fig. 5b left graph; F(1,7) = 80.152; p < 0.00005). By contrast, the reduction in density was less in the SNpc of MPTP mice (−76.1%) compared to vehicle controls (Fig. 5 aII and 5 aIV; 5b right graph; F(1,7) = 157.549; p < 0.00001). As a confirmation, the packing density of all Nissl-stained neurons in nigral structures was quantified within the same anatomic boundaries within which TH-ir cells were counted.

## Discussion

We here present a novel Matlab based script for an in-depth analysis of movement patterns in the traction test. It is inspired by the need to have a high resolution video analysis of the motor coordination voiced by Sedelis and co-workers^10^. It was followed by the development of a classification scheme taking into account that mice execute a selected number of movements when hanging on the underside of a grid^11^. Their classification of fore paw step distance, wall time and fore paw faults was not maintained here as there are numerous intermediates to these criteria (for example: paws go into a hold position between steps and hind paws also shift location as animals move forward). In contrast to Tillerson and colleagues, we therefore started with the Hold position and scored displacement from the location A to location B as a Step. The movements come with numerous parameters (see Tables 1 and 2) and are typically locomotive and executed with all paws, not only front. As a consequence, both the assessment of the movement and the establishment of parameters in our case are unbiased and detailed registration concerns all paws. An interesting and unexpected observation was the fact that short trial times of ≤10 seconds did not provide sufficient data for analysis. This is a clear limitation of the method. However, it should be considered that animals with such short cling times represent a severe motor condition for which the traditional parameter ‘cling time’ will suffice to provide cohort differences.

A strong motivation for translational neuroscience nowadays is the search for novel biomarkers that robustly highlight early onset of diseases. In this context, it is not the severely impaired patient/mouse that is the subject of research, but the subtle differences that reflect small yet progressive changes in neuronal function. Towards this end we did not reveal global gross motor deficits in our MPTP mice despite considerable loss of neurons in the substantia nigra. Nevertheless, our analysis tool *TracMouse* was sensitive enough to return disease relevant deficits.

Our analysis allowed us to explore established and novel, yet unstudied aspects of movement specific to the inverted grid. Hanging upside down is a natural behavior of rodents and can be observed as part of the normal repertoire in their home cages. It therefore does not constitute a significant challenge to a normal mouse and a trial length of 30 s is not sufficient to induce fatigue in most mouse models. After recording of movement at high precision, a distinct two-level approach was implemented: (i) principal identification of movement related anatomical detail in a frame-by-frame manner by a rater blind to the experimental conditions; (ii) rater independent automatic retrieval of parameters and analysis to provide primary endpoints by *TracMouse*. This work plan provides high fidelity between video input and analytical output. It further secures objectivity and enables the mathematical compilation of further high order indices (see Table 2 for some examples). We here concentrated on the primary outcome measures. Overall, there were considerable differences in movement patterns between front and hind paws.

Most of the cling time was spent by each paw holding/gripping on to the wires: forepaws 76%, hind paws 88% hind paws. Add to this the amount of time spent in wall contact (forepaws: 6%, hind paws 8%), it follows that in normal C57BL/6 mice a single paw spent between 82% (forepaw) and 96% (hind paw) stationary simply clinging to the grid. As a corollary, only 18% and 4% of the total cling time was executed as steps by an individual fore and hind paw respectively. In total, this amounts to 44% of the cling time for an animal in step mode and this included times of wall contact. Of particular relevance when concerned with movement is not the period of immobility, but the pattern of locomotion and the execution of movement. However, steps of each paw are conducted only during a very small portion of the total cling time and this readily explains why short trial times invalidate the information that is retrieved for steps.

Since the traction test is considered a task specifically sensitive to forepaw deficits (hence Tillerson and Miller^11^ analysis of forepaws only), it is not surprising that in comparison to hind paws, the forepaws performed almost 4 times more steps and the average duration of the Hold of each forepaw was almost 5 times shorter. Moreover, the average forepaw step duration was about 20% longer and step velocity was two times slower in comparison with hind paw steps. Such a low velocity of forepaw movement resulted in much shorter steps and together with the fact that the distance covered by forepaws was about 2.5 times greater than that by hind paws provides compelling evidence for planned exploratory movements with fore, but not hind paws.

Overall, a detailed analysis of the forepaw movements revealed the occurrence of: (i) steps that can be interrupted by short touches, during which no grip is executed; (ii) trains of steps in which the same forepaw is moving repeatedly while the contralateral forepaw (and hind paws) is stationary; (iii) alternations of long and short (fast and slow) steps of the same paw. Although the occurrence of touches appears to be a rare event for normal animals, touches occurred as an important proxy in the identification of a disease phenotype (MPTP cohort) and this confirms the importance of this readout. Yet, our data set is too small to allow for a compelling statistical verification of this result. At the same time, touches appear to be clear interruptions of the planned motor program and this is a phenotype related to Parkinsonism^22^. In the past, such subtle deficits related to poor motor planning have been difficult to quantify in animals so that steps with touches may be a novel and exciting proxy for the presumed dysfunction of the balance between direct and indirect striatal pathways^23^.

The fact that our principle starting point for analysis is the Hold stage precludes a direct comparison of data with those obtained by the Tillerson and Miller method^11^,^15^,^18^,^20^.

First, we did not classify steps as correct or incorrect, as we did not assess the quality of hold. This assessment would require the knowledge of the exact motor program to be executed by the animal and the comparison between intended and executed movement. Clearly, this is incompatible with a purely behavior based analysis. Nevertheless, the definition of slips as introduced by Tillerson and Miller^11^ as an endpoint with relevance to Parkinsonian deficits (see also refs 16, 17, 18, 21) is somewhat similar to touches introduced here. While it is unclear whether slips are poorly executed attempts to grip the wire, it seems obvious to us that touches are actions for stabilization of the movement and help in orientating the paw towards its destination. They therefore seem to be qualitatively different.

Second, we did not attempt to classify movements into locomotive and non-locomotive. By contrast, we consider all movement as Steps thereby avoiding the introduction of a specific quality. It is not inconceivable that steps with touches are similar to non-locomotive movements. However, we found these to be extremely rare in control mice suggesting that under non-disease conditions, the majority of steps executed by fore and hind paws are locomotive.

Third, Tillerson and Miller^11^ introduced percent wall time as a measure of physical contact with the wall by either the head or the trunk of the body. Despite careful observation, we did not identify such events (also not in the MPTP cohort), but observed paw contacts with the wall. Such wall contacts appear to present a specific form of Hold as they can be long-lasting and include both fore and hind paws.

Forth, we here lay out a series of indices, that can be derived from the observation of Hold, Step, Touches and Wall contacts (see Tables 1 and 2). Clearly these proxies are not exhaustive but introduce some novel means that are helpful in the identification of movement patters in the traction test and may be suitable for the identification of Parkinsonian motor impairments. For example, the heightened number of steps performed by forepaws relative to hind paws suggests that forepaw movement is more exploratory and hind paw movement is more locomotive. This may be converted into an Exploration index and one may predict this to be specific for gender or mouse species, and to be abnormal in models of motor dysfunction.

Our high resolution analysis of movement along the underside of a grid using the *TracMouse* script yielded several statistically significant effects of MPTP treatment. Only a small cohort of mice was tested for feasibility and sensitivity of method, but more routine studies will follow in the future. Clearly, our histopathological analysis confirmed the significant lowering of striatal dopamine and loss of substantia nigra neurons as pertinent for Parkinsonian models^24^,^25^,^26^. This resulted in a heightened number and distance covered by forepaw movements relative to controls. It is suggestive of hyperactivity and therefore in agreement with findings obtained in MPTP mice in the open field^27^,^28^, but appears to be dependent on the method of detection^20^. Moreover, the occurrence of Steps with Touches, an otherwise rare event in normal controls, is of significance for both the MPTP model as it reflects a deficit in motor planning, and the *TracMouse* script enables for the first time to detect such a parameter. Consequently, the routine use of *TracMouse* for experimental models of movement disorders such as amyloid lateral sclerosis (ALS) or motor neuron disease (MND) may lead to the emergence of further disease relevant phenotypes based on high resolution analysis in the traction test.

## Conclusions

The traction test also known as inverted grid test is a popular method to determine movement-related deficits in mouse models of motor diseases. Widely used is the manually accessible cling time although a more sophisticated analysis has been proposed in the past^11^. We here present a novel Matlab based script termed *TracMouse*, which aids in an automatic analysis of movement related features once the anatomical details of phalanges and their spatial arrangement have been annotated by the experimenter. This has led to a number of novel proxies with the state of Hold (no movement) becoming the principle and dominant state. Specific forms of Hold are Wall contacts in which paws make contact with the grid enclosing walls. We further identified exploratory forepaw Steps, which rarely included touches. Touches increased in our MPTP mice as an index of poor motor planning. Hind paw movements were locomotive. These principle parameters may be converted into numerous static and dynamic endpoints and provide a novel platform for in-depth analysis of movement in normal and disease models.

## Methods

### Animals and treatment

Male, 3-month-old, C57BL/6 mice bread at the Medical University of Bialystok (Poland) and delivered to the Nencki Institute 1 month prior to experiments were used. Animals weighed 25–30 g at the beginning of the study and remained sedentary throughout the test. Mice were housed four to six animals per cage with dimensions of 43 cm length, 30 cm width and 15 cm in height filled with sawdust (LIGNOCEL Select Fine, Hygenic Animal Bedding), food (Labofeed B rodent diet, Wytwornia Pasz “Morawski”) and water (normal domestic supply, controlled by Epidemiological Station of Mazowieckie Province Governor) available *ad libitum*, in a holding facility at constant temperature and humidity (23 ± 1 °C, 55 ± 5%) on a 12-h light-dark cycle (lights on: 8.00 am).

A total of 10 animals were used in this study. Animal husbandry was provided by the Nencki Institute animal care facility. All experiments were conducted with the approval of the First Warsaw Local Ethics Committee for Animal Experimentation (Permission No. 347/2012) and carried out in accordance with Polish Law on the Protection of Animals and National Institute of Health’s Guide for Care and Use of Laboratory Animals (Publication No. 85-23, revised 1985) and the European Communities Council Directive (63/2010/EU).

The acute experimental MPTP schedule was applied^29^. Six C57BL/6 mice were injected four times with MPTP hydrochloride (20 mg/kg in saline, intraperitoneally; Axon Medchem, Cat.No.1075) at 2-h intervals within a day. Four saline treated C57BL/6 mice served as control. The traction test was performed on days 10 and 20 post-treatment and tissue harvest commenced after the second behavioral measurement.

### Traction test (Inverted grid test): Apparatus and recording

A green wire mesh grid (12 × 12 cm) with 25 mm^2^ openings surrounded by opaque Perspex walls, 9 cm high, was utilized. Each mouse was placed in the center of the grid and it was turned upside down so the mouse was hanging and clinging to the grid. The distance between grid and floor was 24 cm and soft padding was provided to mitigate falling down. The maximal ‘cling time’ was set to 30 seconds during which subjects were freely moving on the underside of the grid. A trial was terminated once the mouse fell off the grid or it clang to the grid over 30 seconds.

Each test session (on day 10 and on day 20 post-treatment) consisted of 3 trials with an inter-trial interval of 1 minute. An overhead CCTV camera (Sony α37) recorded the trials at 25 Hz and Mpeg files were written to the hard drive of a PC running Windows 7.

### Movement detection and auto-analysis using MATLAB script (*TracMouse*)

Since each test session consisted of 3 trials, our analysis concentrated on the trial with the longest total **Cling Time**. Videos of the selected trial of each mouse were imported into MATLAB (MathWorks, Massachusetts, U.S.A.). We developed a script *TracMouse* with a user interface for manual tagging of video items frame-by-frame. After calibration to the external reference frame of the grid (12 × 12 cm) for the extraction of positional data (all given in mm), the rater blind to the animal’s treatment tagged each individual paw (front left, FL; front right, FR; hind left, HL; hind right, HR) with their phalanges, and each individual event related to movement of the paws (for further details see below; time required: ca. 20 minutes). Our *TracMouse* script enabled continuous tracking of all paw markings simultaneously over time (30 seconds; frame by frame) with subsequent storage of data and export to Excel. The description of movement of each paw was based on three principal components: Hold, Step with or without Touch(es), and Wall Contacts (for definitions, see below). Primary metrics that were extracted from the videos included paw location and timing of events (start, stop). The automatic analysis of *TracMouse* provides highest accuracy with respect to onset and termination of specific movement-related events and average timings/occurrences.

**Figure 6:**
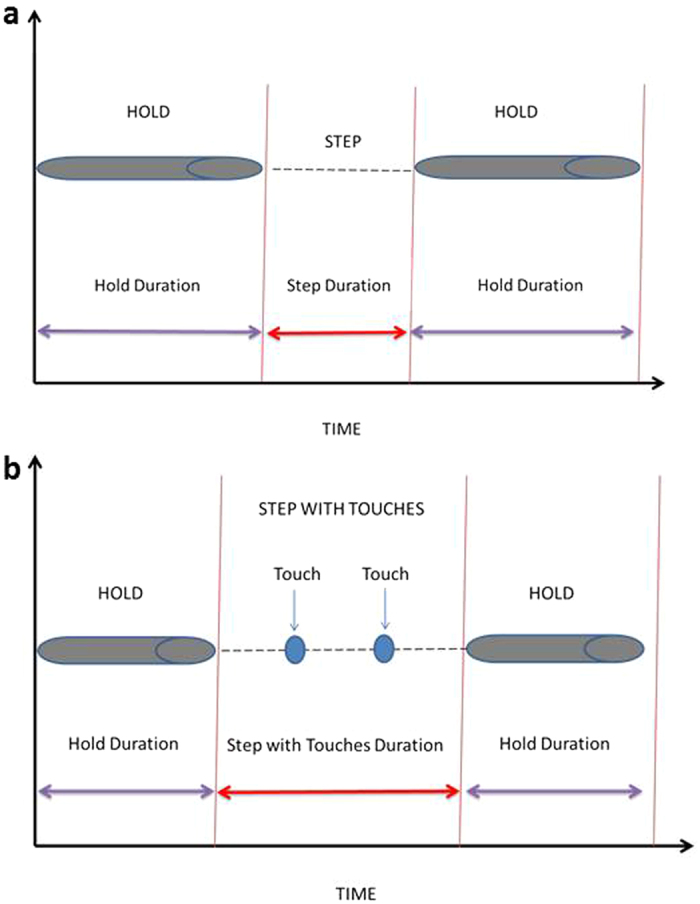
Schematic illustration of the time relations between states defined for each paw. **(a) Hold** occurs when the grid wire is clearly seized with the digit of each individual paw. The rater determines the start/end of each hold during a frame-by-frame identification process. Hold duration then is calculated by *TracMouse*. **Step** is defined as displacement of the paw from the given Hold location to the location of the next Hold. All Step time variables are calculated by *TracMouse* based on variables of preceding and following Holds. **(b)** A **Touch** occurs when the mouse clearly stops the forepaw movement in the immediate vicinity of the wire and touches it, but does not grip. The occurrence of a Touch is determined by the rater. Touches are characterized by their length and frequency of appearance. **Step with Touches** is defined as time between holds in which at least one Touch occurs.

We here follow a hierarchical top-down analysis with the most crude and global data first, from which further refinements were categorized. This principal classifier was constructed from the basic spreadsheet software and resultant output included the traditionally applied **Cling Time** before fall off. A second metric concerned the behavioral state of the paw, for which we prescribed the binary decision as belonging to **Hold** or **Step** (Fig. 6a). **Hold** is defined as all paw digits seizing the grid wire with XY coordinates remaining constant; for each individual paw the output classifier is **Hold duration** as the sum of durations of all Holds during a trial, as well as the **Total number of Holds**. **Step** in its most simple category is defined as a paw displacement from the given Hold position to a new Hold in a different location (displacement of paws and return to original position were not counted as steps). The primary output for individual paws includes **Total number of Steps without Touches**, **Total Step Distance** – i.e. distance covered by a given paw during all steps in a trial, and **Step duration** – i.e. total time in stepping mode during the trial. A more complex form of step includes intermediate touches (Fig. 6b). **Touch** occurs during steps when the movement slows down and the paw (typically front paw) makes contact with the wire without performing a grip. Based on the frame-by-frame analysis, the velocity of paw movement at the beginning of steps is high and slows down towards touches or towards the end of the step when a grip is immanent. Steps were observed to have single or multiple touches. In case of Touches and Steps with Touches, due to their rare occurrence the primary (and final) output includes only **Total number of Touches** and **Total number of Steps with Touches**. **Wall Contacts** are observed during steps when a paw touches the walls surrounding the grid. These contacts are infrequent, typically long-lasting and, similar to holds, interrupt the step and the propensity of the animal to move forward. From the frame-by-frame analysis algorithm, **Total number of Wall Contacts** and **Wall Contact duration** – i.e. total time spent in Wall Contact by individual paws, are extracted.

**Figure 7:**
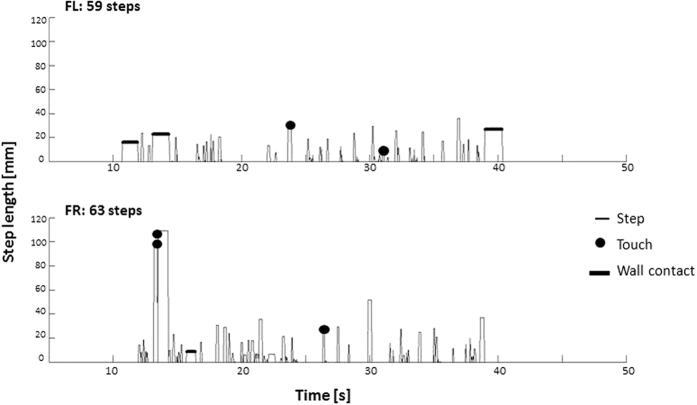
Examplar step histograms for left (FL) and for right forepaw (FR) of the same mouse in the same trial. Apart from the alternative use of the left and right forepaw, we observed short series of consecutive steps performed with the same paw (termed Trains). Also, multiple episodes of Wall Contacts and Touches took place–there were two Steps with single Touch performed by left forepaw, one Step with double Touch and one Step with single Touch performed by right forepaw.

Although not explored in great detail here, a differentiated sequential register of individual events (Holds, Steps, Touches, Wall Contacts) can be extracted on demand. Figure 7 depicts two exemplar traction test histograms of a representative control mouse with differential activity of the left and right forepaws (FL and FR). No activity indicates the state of Hold, which dominates in duration; upward deflections indicate Steps and in few cases Touches and/or Wall Contacts are also marked. It should be intuitively clear that this pattern is intractable using the standard stop-watch observation method for the traction test.

**Figure 8:**
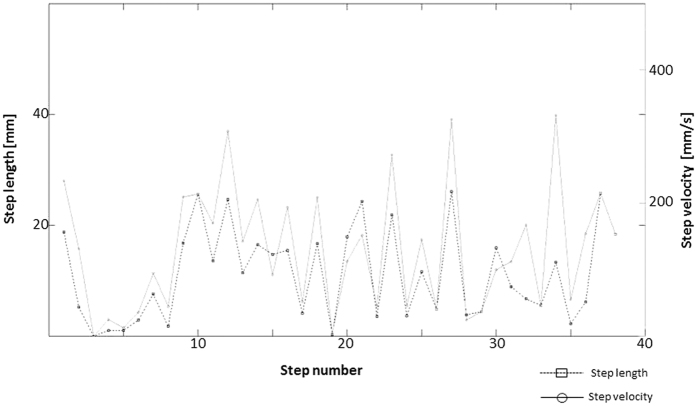
Changes in step length accompanied by changes in step velocity; long steps are performed with higher velocity than short ones. Note the alternation of long-short-long step sequences with one given paw. Such sequences can be used to calculate a regularity index reflecting the overall amount of alternation between long and short steps (see Fig. 7).

All primary proxies have been normalized in such a way, that they became related to nominal 30 seconds trial. This was achieved by dividing their values with Cling Time and multiplying by 30 seconds. Once primary proxies were extracted and normalized, a further characterization of the categories of movement was attained through calculation of secondary indices (see below and Table 1). Normalization did not affect these proxies which were defined as ratios of primary endpoints. The parameter definitions are collected in Table 2. Several secondary calculations were performed based on the principal output of *TracMouse*, but there were also some additional features that only became obvious by plotting the acquired data sets. As may be seen in Fig. 7, both left and right forepaw either alternated i.e. subsequent steps were performed by left and right paw in turn, or few subsequent steps were performed by the same paw. Repeated step sequences of the same paw were termed **Trains**. However, for calculation of **Number of Trains** and **Steps per Train,** we plotted the number of steps performed during a single trial (abscissa) against their length and velocity (ordinate) (Fig. 8). It emerges that short and long (slow and fast) steps seem to alternate. From this observation, we derived a **Regularity Index** by counting the number of peaks (long steps) in relation to the total number of steps. The Regularity Index can take values from 0 to 1, where a value of 1 denotes repetition of short step – long step sequences performed by the same paw throughout the whole trial. Note that this regular stride pattern is not continuous as it is interrupted by steps made by the opposite paw. Intriguingly though, the step-length alternation remains stable for each forepaw. A similar Regularity Index could be derived for step velocity.

### Perfusion and processing of mouse brain

Mice were deeply anesthetized with Vetbutal and intraaortic perfused with cold (4 °C) phosphate buffered saline (PBS) containing 5 IU of heparin per 1 mL of buffer (50 ml within 5 minutes) followed by 4% paraformaldehyde in PBS (fixative, 50 ml within 5 minutes) and subsequent 5% glycerol and 2% dimethyl sulphoxide (DMSO) in PBS (50 ml within 5 minutes). The brains were removed, placed for 1 hour in fixative and then immersed for cryoprotection in 10% glycerol and 2% DMSO (24 h) and subsequently in 20% glycerol and 2% DMSO (24 h). For immunohistochemical analysis, brains were coronally sectioned at 40 μm thickness with a freezing stage microtome (Leica CM1850). Consecutive sections were collected throughout the forebrain and midbrain and stained for Nissl and immunolabeled with antibody against tyrosine hydroxylase.

### Tyrosine hydroxylase (TH) immunohistochemistry

Free-floating sections from frozen mouse brains were first incubated in 1% solution of H_2_O_2_ in 0,1 M PBS for 30 min to reduce activity of endogenous peroxidase, blocked with 5% solution of normal goat serum (NGS) in 0.1 M PBS-T (PBS with 0,3% Triton) for 60 min. This was followed by incubation in anti-tyrosine hydroxylase antibody (AB152, Merck Millipore, Massachusetts, U.S.A.) solution (diluted 1:1000) in 0.1 M PBS-T with 5% NGS and 1% bovine serum albumin (BSA) for 1 hour at RT and 19 hour at 4 °C. The next day the sections were treated with goat anti-rabbit biotin conjugated IgG solution (Vector Laboratories, BA-1000; diluted 1:200) in 0,1 M PBS-T with 5% NGS and 1% BSA for 1 hour at room temperature (RT) and then incubated for 1 hour at RT with peroxidase conjugated with streptavidin (diluted 1:500, Vector Laboratories, PK-4000). To visualize primary-secondary antibody complexes sections were incubated for 5 minutes in PBS containing 3,3′-diaminobenzidine tetrahydrochloride (DAB, Sigma-Aldrich, D8001) as a chromogen and H_2_O_2_ at a concentration of 0.05% and 0.01%, respectively. The stained sections were mounted, air dried and cover slipped. Sections stained with DAB were analyzed using a bright field microscope (Nikon Eclipse Ni-E) equipped with the Nikon DS-Ri2 camera and NIS image analysis software. Controls for the immunohistochemical procedure were obtained running some slides through the entire procedure with the omission of the primary or secondary antibodies as a safeguard against nonspecific staining by the primary antibody. No immunoreactivity was observed for any control slides (data not shown).

### Computer-assisted densitometric analysis of TH-ir neurons

In order to establish whether dopaminergic neurons were lost, series of sections from 6 MPTP and 4 control brains were selected. Sections were viewed with a Nikon Eclipse Ni-E microscope equipped with an x/y movement-sensitive stage and video camera attached to an IBM PC Pentium. A computerized image analysis system (NIS-Elements, Nikon Instruments; digitizer and software) was used for densitometric analysis of TH-ir neurons in the ventral tegmental area (VTA) and in the substantia nigra pars compacta (SNpc). Brain regions were identified based on the mouse brain atlas^30^. The boundaries of the structures in the coronal plane were determined microscopically and marked on line drawings using a computer-aided X-Y plotting system. The system calculated the square area [mm^2^] of the counting frames. Subsequently, counting of the number of TH-ir neuronal profiles in each outlined area was done at 400x magnification. Quantitative analysis was performed by using the criteria that neuronal somata were TH-immunoreactive and the cell nucleus and proximal segment of one or two dendrites were well visible within the counting frame. These criteria enabled exclusion of non-complete remnants of neurons. Neuronal counts were performed by two independent observers (inter-observer correlation *r* = 0.87). Sections were selected for SNpc or VTA corresponding to Bregma −3.15 and −3.51 (SNpc) and Bregma −2.79 and −3.07 (VTA) respectively. Counts per section were corrected by Abercrombie’s^31^ formula. Afterwards, the packing density (PD) of dopaminergic neurons was calculated as a function of rostro-caudal level and of location within VTA and SNpc by using the determined number of cells and square area of outlined frames in each section analyzed. The following equation was used:


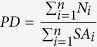


where, PD - mean packing density [mm^−2^]; N_i_ - number of counted neurons in i-th section (corrected by Abercrombie’s formula); SA_i_ - square area of i-th analyzed frame [mm^2^]. Thus, the mean number of TH-ir neurons found in each experimental group was expressed as packing density (number of cells per one square millimeter).

### Statistical analysis

All data were expressed as mean ± SD and compared by Analysis of Variance using within and between subject factors followed by multiple range Newman-Keuls *post-hoc* test. The analysis was made using STATISTICA 10 software with alpha set to 5%. Only significant terms are mentioned in the text for clarity. A complete summary of the statistical comparisons between treatment groups and paws is displayed in Table 4.

## Additional Information

**How to cite this article**: Niewiadomski, W. *et al*. TracMouse: A computer aided movement analysis script for the mouse inverted horizontal grid test. *Sci. Rep.* **6**, 39331; doi: 10.1038/srep39331 (2016).

**Publisher’s note:** Springer Nature remains neutral with regard to jurisdictional claims in published maps and institutional affiliations.

## Figures and Tables

**Table 1:** Hierarchical organization of analysis parameters calculated using *TracMouse* from video-data of the inverted grid.

*Category (Mode)*	*Identifier*		*Primary Proxy*	*Secondary Proxy*
	*Time*	*Coordinates*	*(for each paw)*	*(for each paw)*
	StartStop		- Cling Time*(whole animal)*	
Hold	StartStop		- Total number of Holds- Holds duration	- Mean duration per Hold
Step without Touches	StartStop	XY startXY end	- Total number of Steps without Touches- Steps duration- Steps distance	- Mean Step duration- Mean Step length- Mean Step velocity- Number of Trains- Steps per Train- Regularity Index
TouchStep with Touch(es)	Time of occurrenceStartStop	XY startXY end	- Total number of Touches- Total number of Steps with Touches	
Wall Contact	StartStop		- Total number of Wall Contacts- Wall Contacts duration	

The table shows the principle metrics extracted from the time histograms in a frame-byframe manner.

After definition of the overarching behavioral Categories and their identification on the video, Primary and Secondary proxies for each paw were calculated.

For definition of parameters, see Material and Methods and Table 2.

**Table 2:** Definitions of primary and secondary proxies from the *TracMouse* system.

*TracMouse parameters*	*Definition*
**Cling Time**	**Overall time in which animal kept contact with a grid (latency to fall; max 30 s)**
**Hold**	**Time with all digits seizing the grid wire and XY coordinates being constant**
Total number of Holds*	Total number of Hold episodes during the trial
Holds duration *[s]	Total time in Hold during the trial
Mean duration per Hold [s]	Holds duration/Total number of Holds
**Step without Touch**	**Paw displacement from given XY coordinates of a given Hold to new XY coordinates of the next Hold;(if a Step includes a Touch see below)**
Total number of Steps without Touches*	Number of completed Steps during the given trial without Touches
Steps duration*[s]	Cumulative time spent in Step mode during trial^a^
Steps distance*[mm]	Cumulative distance of all Steps during trial^a^
Mean Step duration [s]	Steps duration/Total number of Steps without Touches
Mean Step length [mm]	Steps distance/Total number of Steps without Touches
Mean Step velocity [mm/s]	Step distance/Total number of Steps without Touches
**Touch**^b^ **Step with Touch(es)**^b^	**During Step, a paw makes contact with grid; however the phalanges do not perform a grip but the paw moves on to a different XY coordinate or returns to its original location (exploratory steps with touches)**
Total number of Touches*	Total number of Touches during trial
Total number of Steps with Touches*	Total number of Steps with one/multiple Touches
**Wall Contact**	**A paw makes contact with the wall**
Total number of Wall Contacts*	Total number of Wall Contacts per trial
Wall Contacts duration*	Cumulative time spent in Wall Contact mode during trial
**Train**	**Series of Steps (≥2) of the same forepaw**
Number of Trains*	Number of occurrences of Trains per trial
Steps per Train	Total number of Steps without Touches /Number of Trains
Regularity Index	The number of peaks (local maxima) in the time course curve of Step length, where on X axis no. of Step, on Y axis its length in mm, divided by ½ (Total number of Steps without Touches -1)

Note that most of these parameters can be individually calculated for each paw.

Explanations:

^*^Denotes parameter normalization i.e. division by Cling Time and multiplication by nominal length of the trial i.e. 30 s.

^a^Only Steps without Touches.

^b^Only applies to forepaws.

**Table 3:** Cling Time for each individual included in this study and the respective correction factor.

Treatment	Cling Time Session 1	Correction Factor	Cling Time Session 2	Correction Factor
***Control***	31.28	0.9591	31.44	0.9542
***Control***	32.32	0.9282	15.40	1.9481
***Control***	32.08	0.9352	31.24	0.9603
***Control***	31.80	0.9434	31.80	0.9434
	*Mean* ± *S*D 31.87 ± 0.45		*Mean* ± *SD* 27.47 ± 8.05	
***MPTP***	32.04	0.9363	31.40	0.9554
***MPTP***	32.64	0.9191	30.56	0.9817
***MPTP***	31.16	0.9628	16.20	1.8519
***MPTP***	33.00	0.9091	31.20	0.9615
	Mean ± SD 32.21 ± 0.80		Mean ± SD 27.34 ± 7.44	

The Cling Time was recorded as – the longest period of clinging to the underside of the inverted grid out of 3 consecutive trials in Session 1 performed 10 days after saline (Control) or MPTP treatment and in Session 2 performed 20 days post-treatment. Note that the time it took to stop the video recording and remove the mouse exceeded 30 seconds. We normalized this value for all mice to introduce greater homogeneity into the data set and to avoid inaccuracies in the frame cut-off during TracMouse analysis. The data provide compelling evidence that 30 seconds is readily sustainable for both control and MPTP mice and that Cling Time is not a differential proxy for these cohorts. (It may become a differential for Parkinsonian models if extended for longer periods). *Not included 2 MPTP mice, because of very short Cling Time in Session 1 (see text). Correction Factor is equal 30 s/Cling Time.

**Table 4:** Summary Table detailing the statistical differences between treatment and paw axes for all proxies that are presented in Figs 1, 2 and 3.

Proxy	Figure	Significance
Cling Time	1a	Treatment: NSSession: NSInteraction: NS
Total number of Holds	1b	Treatment: NSFront v Hind: F(1,6) = 504, p < 0.00001*Interaction: F(1,6) = 7, p < 0.05*
Holds duration	1c	Treatment: NSFront v Hind: F(1,6) = 51, p < 0.0005Interaction: NS
Mean duration per Hold	1d	Treatment: NSFront v Hind: F(1,6) = 40, p < 0.001Interaction: NS
Total number of Steps without Touches	2a	Treatment: NSFront v Hind: F(1,6) = 335, p < 0.00001Interaction: NS
Total distance of Steps	2b	Treatment: NSFront v Hind: F(1,6) = 177, p < 0.00005*Interaction: F(1,6) = 11, p < 0.05(forepaws of control v MPTP mice: p < 0.05; hind paws: NS)*
Total duration of Steps	2c	Treatment: NSFront v Hind: F(1,6) = 284, p < 0.00001Interaction: NS
Mean duration of a Step	2d	Treatment: NSFront v Hind: F(1,6) = 43, p < 0.001Interaction: NS
Mean length of each Step	2e	Treatment: NSFront v Hind: F(1,6) = 129, p < 0.00005Interaction: NS
Mean Step velocity	2f	Treatment: NSFront v Hind: F(1,6) = 119, p < 0.00005Interaction: NS
Total number of Touches	3a	Not Analyzed (see text for details)
Total number of Steps with Touches	3b	Not Analyzed (see text for details)
Total number of Wall Contacts	3c	Treatment: NSFront v Hind: NSInteraction: NS
Wall Contacts duration	3d	Treatment: NSFront v Hind: F(1,6) = 7.74, p < 0.05Interaction: NS
